# Face Mask Use and Associated Factors Among Students: Mixed Methods Study

**DOI:** 10.2196/41365

**Published:** 2023-05-22

**Authors:** Abreha Addis Gesese, Tut Duer Thot

**Affiliations:** 1 Department of Clinical Nursing Gambella Teachers Education and Health Science College Gambella Ethiopia; 2 School of Public Health College of Health and Medical Sciences Haramaya University Harar Ethiopia

**Keywords:** face mask use, associated factors, COVID-19, Gambella, students, Ethiopia

## Abstract

**Background:**

COVID-19 has gravely affected the world, including students, due to the high level of contracting infections.

**Objective:**

This study assessed the magnitude of mask use and associated factors among students.

**Methods:**

A cross-sectional study using mixed methods was conducted among students at Gambella Teachers’ Education and Health Science College, Gambella Region, Southwest Ethiopia, from March 5 to March 30, 2021. The stratified random sampling technique was used. Proportional allocation of samples was used to randomly select case teams, and a simple random sampling technique was used to recruit the students. The data were collected by trained and experienced enumerators. Data were entered into EpiData (version 3.1; EpiData Association) and exported to SPSS (version 22; IBM Corp) for analysis. Logistic regression was executed. The adjusted odds ratio (AOR) with the 95% CI was used to determine the association and strength with the outcome variable. The qualitative data were transcribed, translated, coded, and analyzed using thematic analysis. Then, the themes were used to triangulate the quantitative study.

**Results:**

The study included a total of 379 participants and yielded a response rate of 95.5% (379/397). The majority of study participants were older than 25 years, with the mean age being 26.34 (SD 5.8) years. This study found that the magnitude of mask use among students was 87% (330/379). The odds of mask use were higher among students who were female (AOR 3.32, 95% CI 1.191-9.248), younger (AOR 2.55, 95% CI 1.155-5.627), agreed that not all persons with COVID-19 develop severe disease (AOR 3.38, 95% CI 1.36-8.41), agreed that there is currently no effective cure (AOR 6.28, 95% CI 1.36-28.99), performed proper washing with soap and water (AOR 0.027, 95% CI 0.004-0.182), had started to stay home (AOR 0.168, 95% CI 0.054-0.52), agreed that COVID-19 is fatal (AOR 0.236, 95% CI 0.084-0.666), agreed that a flu vaccine is sufficient for COVID-19 prevention (AOR 3.874, 95% CI 1.540-9.749), and disinfected equipment and working areas at least once a day (AOR 0.222, 95% CI 0.086-0.575).

**Conclusions:**

This study found that the magnitude of mask use among students was relatively moderate in Ethiopia. Sex, age, agreeing that not all persons with COVID-19 develop severe disease, agreeing that there is currently no effective cure, performing proper washing with soap and water, starting to stay home, agreeing that COVID-19 is fatal, and agreeing that the flu vaccine is sufficient to prevent COVID-19 were independently associated with mask use among students. Therefore, colleges should aggressively encourage students to wear masks and monitor the implementation of COVID-19 prevention regulations along with the accessibility of masks.

## Introduction

COVID-19 is a respiratory tract infection and a public health emergency of international concern [1]. It is epidemiologically associated with the Wuhan, Hubei Province, Seafood Wholesale Market, where birds, bats, snakes, and other wildlife animals are sold [2]. COVID-19 differs with respect to community spread and severity [3]. After exposure to the virus and an incubation period of 2 to 14 days, people with COVID-19 develop a wide range of symptoms, which have been reported to range from mild to severe illness and include fever or chills, cough, shortness of breath or difficulty breathing, fatigue, muscle or body aches, headache, loss of taste or smell, sore throat, congestion or runny nose, nausea or vomiting, and diarrhea. Patients with COVID-19 can experience only mild or uncomplicated illness, and approximately 14% develop a severe illness that requires hospitalization and oxygen support, with 5% requiring admission to an intensive care unit [1,4]. COVID-19 can be transmitted through droplets, direct and indirect contact, and aerosols in long-range transmission (ie, airborne transmission) [5].

Globally, as of June 28, 2021, there had been 182,037,151 confirmed cases, 3,942,149 deaths, and 166,525,346 recovered cases of COVID-19 reported to the Worldometer [6]. The United States, India, and Brazil were among the countries with the highest confirmed cases in 2021 [6-8]. In Africa, approximately 3,942,448 infected patients in 47 countries led to a cumulative 94,217 deaths [6]. In Ethiopia, after the first report of a COVID-19 case, more than 276,000 confirmed cases and 4300 deaths have occurred [8].

Rigorous trials have brought COVID-19 vaccines, although these have side effects and quality differences, and they are the subject of popular myths [9,10]. However, there is lack of effective and approved drugs to treat COVID-19 in certain countries. Instead, strategic efforts have focused on combatting the spread of the disease. Recommendations have been made to prevent the most contagious viral diseases; one of these is the use of face masks. This is the recommendation to the general public that they should wear nonmedical masks in indoor settings (eg, shops, shared workplaces, and schools) and outdoor settings where physical distancing is not possible to a minimum interval of 1 meter [11]. The universal use of face masks can contribute to the containment of the virus in the community if they are adequately available, properly used, and properly disposed of after use [12]. The absence of clear scientific evidence for aerosol transmission of SARS-CoV-2 provides the rationale for the current recommendations for the use of surgical masks among health care professionals. There are also other means of preventing COVID-19, such as frequent hand washing with soap and sanitizer [13]. A study from the United Kingdom suggested that making masks mandatory in secondary schools would be of benefit but would need to be combined with scaling up of test-trace-isolate coverage to prevent resurgence of COVID-19. We highlight that the adoption of masks in schools, in addition to community settings, can help reduce epidemic resurgence but that to do this effectively, access to masks has to be sufficiently high. Studies show that if access is lower, the estimated reduction in COVID-19 resurgence is smaller. Uncertainties concerning the effectiveness of masks remain, and these results add to the ongoing body of evidence on the impact of using face masks to combat epidemics [14]. Face mask use could result in a large reduction in the risk of infection, with stronger associations with N95 and similar respirators than with disposable surgical masks [15,16].

Despite the benefits of face mask use, studies have reported that face mask use varies across different settings. A study from Japan reported that most respondents showed a moderate or higher frequency of washing their hands or wearing masks (both at 96.4%) [17], while in Saudi Arabia, the majority (91%) reported that they were following the strategies recommended by the authorities to prevent the spread of the virus [18]. In Mizan-Tepi, Ethiopia, the majority (55%) did not wear face masks [19], while in the United Arab Emirates more than 90% did so [20]. Positive correlations were discovered between attitude and practice [21] among primary school students in Wuhan, China (51.6%) [22], in western Uganda (95.2%) [23], and in Dessie and Kombolcha towns in Ethiopia (74.1%) [24].

After the launch of safe school reopening programs in Ethiopia [25], there was high demand for prevention and control efforts to be strengthened. Students are among at-risk groups for contracting COVID-19, which in turn can affect a large number of families and communities. Knowing this will help to set priorities and design effective and consistent preventive measures at school. Thus, this study aimed to assess the level of mask use and associated factors among students at Gambella Teachers’ Education and Health Science College, Gambella, Ethiopia, in 2021.

## Methods

### Study Area and Period

The study was conducted in Gambella Teachers’ Education and Health Science College, Gambella Region, Southwest Ethiopia, from March 5 to 30, 2021. This college is located in Gambella Town, 766 km away from Addis Ababa, the capital city of Ethiopia. The region has a total population of 435,999 [26]. After the report of the first COVID-19 case in Ethiopia, the government, in collaboration with responsible bodies, implemented strict COVID-19 prevention and control measures. Despite that effort, the number of infected patients increased because of limited capacity and access to services. Gambella is one of the regional states that were among the settings of the COVID-19 program. As part of this program, a total of 5 hospitals and 148 other health institutions and isolation centers provided services all over the region. The college provides teaching and learning to regular, private, and extension-program students. A total of 1041 regular, 1262 summer, and 2563 extension students were enrolled from the teaching stream and 659 regular and 2256 private students were enrolled from the regular health stream. Of these students, 2763 were men and 1515 were women. The college also provides scholarship programs for students from South Sudan and refugees in partnership with various nongovernmental organizations [27].

### Study Design

This was a cross-sectional study using mixed methods.

### Source Population and Study Population

The source population included all students in the Gambella Teachers’ Education and Health Science College during the study period. However, the study population included only students in selected streams and departments during the study period.

### Inclusion and Exclusion Criteria

All active students registered in the second semester of the 2021 academic calendar year or later academic years were included in this study. Students who were seriously ill or absent during the data collection period were excluded from this study.

### Sample Size Determination and Sampling

The sample size was determined using a single-population proportion formula with the following assumptions: 95% CI, 5% type I error, and a proportion of respondents whose attitude level was 56.4%. Finally, the researchers added 5% to compensate for the nonresponse of participants, making the final sample size 397 after considering other objectives.

A stratified random sampling technique was used. First, the college was stratified into teachers’ education and health science streams based on academic setting. This college has 2 broad streams (health and teaching). Among academic departments, 5 are health departments and about 20 are teachers’ streams or departments. Then, the sample was proportionally allocated to size. Finally, a simple random sampling technique was used to enroll 1 study participant from a health stream to every 4 participants from teaching streams. Purposely selected participants were interviewed for the qualitative study.

### Data Collection Instrument and Procedure

A pretested and translated version of a self-administered questionnaire was used for data collection. This questionnaire measured sociodemographic characteristics, knowledge of COVID-19 infection and transmission, attitude-related variables, magnitude, and associated factors. Data collection tools were gathered and adapted from previous research [9,17,19,28]. The data were collected by trained and experienced enumerators. Two days’ training was provided to the data collectors and supervisors on the study purpose and methodology and on how to conduct and administer the self-administered questionnaire, take consent, keep confidentiality, and respect the rights of the participants. A minimum of a 1-meter distance was kept between interviewers and interviewees. A semistructured interview guide was used for collecting qualitative interviews. The interviewer used taped records and took notes. Interviews lasting a minimum of 30 to 45 minutes were conducted with the study participants. The research was conducted after the lockdown.

### Data Processing and Analysis

The collected data were first checked manually for completeness and consistency at the time of data collection and then rechecked at the office by the principal investigator before data entry. Then, the data were entered into EpiData (version 3.1; EpiData Association) and exported to SPSS (version 22; IBM Corp) for analysis.

Descriptive statistics are reported for sociodemographic characteristics and knowledge, attitude, and practice variables as the mean (SD) and range for numerical data and frequency and percentage for categorical data, including the magnitude of mask use.

Then, a logistic regression analysis was performed to determine the strength of associations with the outcome variable using the adjusted odds ratio (AOR) with the 95% CI. The final model fitness was checked using Hosmer-Lemeshow goodness of fit. The qualitative data were transcribed, translated, coded, and analyzed using thematic analysis. Then, the themes were used to triangulate the quantitative study.

### Data Quality Management

Data collectors were trained on the data collection tool contents and how to collect them. A pretested questionnaire was used on 5% of respondents from another nearby college. The completeness, accuracy, and consistency of the collected data were checked by the principal investigator. Data were edited, coded, and entered into a computer. Then, computer data cleaning was performed to check for the consistency of data.

### Operational Definition

Face masks were defined as disposable or reusable devices that create a physical barrier between the mouth and nose of the wearer and potential contaminants in the immediate environment.

### Ethical Approval

Ethical approval was obtained from Gambella Teachers’ Education and Health Science College Research Ethics Review Committee (registration number: GTE/939/355/2020). Verbal and written informed consent was obtained for the quantitative and qualitative data, respectively. The informants were assured that all written and recorded data would be kept confidential by using codes to identify participants instead of names or any other personal identifiers. Informants were clearly informed about their right to refuse to participate in the study or withdraw at any time during the interview session.

## Results

### Sociodemographic Characteristics

The study included total of 397 participants with a response rate of 95.5% (n=379). The majority of study participants were older than 25 years, and the mean age was 26.34 (SD 5.8) years. About 86% (327/379) of the study participants were men and 55.1% (209/379) were Protestant. A total of 261 (68.9%) of the participants earned a monthly income between US $9.29 and US $37.26 (Table 1).

**Table 1:** Sociodemographic characteristics of students (n=379) at Gambella Teachers’ Education and Health Science College, Gambella Region, Southwest Ethiopia.

Variable		Values
Age (years), mean (SD)		26.34 (5.8)
**Age group (years), n (%)**		
	<20	88 (23.2)
	20-25	75 (19.8)
	>25	216 (57)
**Sex, n (%)**		
	Male	327 (86.3)
	Female	52 (13.7)
**Marital status, n (%)**		
	Single	151 (39.8)
	Engaged/married	228 (60.2)
**Religion, n (%)**		
	Muslim	27 (7.1)
	Orthodox	48 (12.7)
	Protestant	209 (55.1)
	Catholic or other	72 (19)
**Monthly income (US $), n (%)**		
	Up to 9.30	103 (27.2)
	9.31-37.25	261 (68.9)
	37.26 or more	15 (4)

### Knowledge of COVID-19 Infection and Transmission Among Students

The following sections describe the students’ knowledge on COVID-19 infection according to the quantitative data.

#### Symptoms

A high proportion (360/379, 95%) of the students knew that the main clinical symptoms of COVID-19 are fever, fatigue, dry cough, and myalgia, while 234/379 (61.7%) of the participants mentioned other symptoms, such as stuffy nose, runny nose, and sneezing, which distinguish COVID-19 from the common cold and flu.

#### Risk Factors and Prognosis

A total of 195 of 379 (51.5%) of the students knew that elderly people who have chronic illnesses and obesity are at higher risk of developing a severe form of COVID-19, whereas 83.1% (316/379) knew that COVID-19 has no effective cure, yet seeking early treatment increases the chance of recovery.

#### Mode of Transmission

About 83% (316/379) of the students knew that the COVID-19 virus spreads via respiratory droplets from infected people and 77.8% (295/379) of the students knew that asymptomatic transmission is possible.

#### Knowledge About Prevention

A higher proportion (n=362, 95.5%) of the 379 students knew that proper hand washing with soap and water and wearing of general medical masks by ordinary residents can prevent infection. Similar proportions of participants knew not to touch the eyes or nose with unwashed hands (n=295, 77.8%); that they should avoid going to crowded places, such as train stations and public transportation (n=333, 87.9%); that they should avoid contact with people with a known history of infection (n=317, 83.6%); and that isolation and treatment of people who are infected with COVID-19 are effective ways to prevent the virus (n=311, 82.5%). However, 125 (33%) agreed that children and young adults do not need to take measures to prevent infection with COVID-19. (Table 2)

**Table 2:** Knowledge of students about mode of transmissions and infectiousness at Gambella Teachers’ Education and Health Science College, Gambella Region, Southwest Ethiopia.

Variables (n=379)		Respondents (n=379), n (%)	
		Correct	Incorrect
**Knowledge of symptoms**			
	Main clinical symptoms of COVID-19 are fever, fatigue, dry cough, and myalgia	360 (95)	19 (5)
	Unlike the common cold, stuffy nose, runny nose, and sneezing are less common in persons infected with COVID-19	234 (61.7)	145 (38.3)
**Knowledge of high risk and prognosis**			
	Not all persons with COVID-19 will develop severe cases. Only those who are elderly, have chronic illnesses, and are obese are more likely to be severe cases	195 (51.5)	184 (48.5)
	There currently is no effective cure for COVID-2019, but early symptomatic and supportive treatment can help most patients recover from the infection	316 (83.1)	61 (16.6)
**Knowledge about mode of transmission and infectiousness**			
	COVID-19 spreads via respiratory droplets of infected individuals	316 (83.1)	61 (16.6)
	Eating or contacting wild animals would result in the infection by COVID-19	255 (67.7)	124 (32.7)
	Persons with COVID-19 cannot infect the virus to others when a fever is not present	122 (32.2)	257 (67.8)
**Knowledge about ways of prevention**			
	Proper hand washing with soap and water is one method of preventing COVID-19	362 (95.5)	17 (4.5)
	One way of preventing COVID 19 is not touching the eyes or nose with unwashed hands	295 (77.8)	84 (22.2)
	To prevent infection with COVID-19, individuals should avoid going to crowded places, such as train stations, and avoid taking public transportation	333 (87.9)	46 (12.1)
	Ordinary residents can wear general medical masks to prevent infection by COVID-19	362 (95.5)	17 (4.5)
	People who have contact with someone infected with COVID-19 should be immediately isolated in a proper place	317 (83.6)	62 (16.4)
	Isolation and treatment of people who are infected with COVID-19 are effective ways to reduce the spread of the virus	311 (82.5)	68 (17.9)
	Children and young adults do not need to take measures to prevent COVID-19	125 (33)	254 (67)

### Attitude-Related Variables of Students

Regarding the attitude of participants, 90.8% (344/379) of the students agreed that COVID-19 symptoms appear in 2 to 14 days, 88.7% (336/379) of the participants agreed COVID-19 is fatal, and 67.3% (255/379) of students knew that sick patients should share their recent travel history with health care providers. A total of 227 (59.9%) participants agreed that flu vaccination is sufficient, while 320 (84.4%) agreed that eating well-cooked food can sufficiently prevent COVID-19. About 70% (266/379, 70.2%) of students had a positive attitude toward disinfecting areas at least once a day and 98.4% (373/379) had a positive attitude toward washing hands to help prevent COVID-19 transmission (Table 3).

**Table 3:** Attitude-related variables of students and associated factors at Gambella Teachers’ Education and Health Science College, Gambella Region, Southwest Ethiopia.

Attitude-related variables	Respondents (n=379), n (%)	
	Yes	No
COVID-19 symptoms appear in 2 to 14 days	344 (90.8)	35 (9.2)
COVID-19 is fatal	336 (88.7)	43 (11.3)
Flu vaccination is sufficient for COVID-19	227 (59.9)	152 (40.1)
Eating well cooked food and safely handled meat is safe	320 (84.4)	59 (15.6)
Sick patients should share their recent travel history with health care providers	255 (67.3)	124 (32.7)
Disinfect areas at least once a day	266 (70.2)	113 (29.8)
Washing hands helps prevent COVID-19 transmission	373 (98.4)	6 (1.6)

### Magnitude of Mask Use Among Students

The magnitude of mask use among students was found to be 87% (330/379). The qualitative findings included the following observation: “about half of the students reported that mask use prevents COVID-19 infection; however, they failed to apply the regulation regularly. For this, students reported that lack of access to masks was a challenge during their stay in the college.”

### Factors Associated With Mask Use Among Students

After selecting candidates in a bivariate analysis at *P*≤.25, the following variables were independently associated with mask use in the multivariate analysis using AOR and *P*≤.05: sex, age, agreement that not all persons with COVID-19 develop severe disease, agreement that there is currently no effective cure, adherence to proper washing with soap and water, other variables (rubbing with alcohol and avoiding contact with surfaces), having started to stay home, agreement that COVID-19 is fatal, and agreement that flu vaccination and eating well-cooked food are sufficient to prevent COVID-19.

The odds of mask use among female students were 3.3 times higher than among male students (AOR 3.32, 95% CI 1.191-9.248). Younger students was 2.55 times more likely to use face masks than older students (AOR 2.55, 95% CI 1.155-5.627) and students who agreed that not all persons with COVID-19 develop severe disease were 3.38 times more likely to wear a mask than those who did not (AOR 3.38, 95% CI 1.36-8.41). Students who responded that there was currently no effective cure were 6.28 times more likely to wear a mask than those who did not (AOR 6.28, 95% CI 1.36-28.99).

The qualitative interviews led to the following observation: “the majority of respondents reported that COVID-19 is a serious problem that affects all age groups regardless of their sex, race, level of income and educational status. No cure was affirmed globally; as a result, emphasis should be given to prevention and control.”

Students who reported that they performed proper washing with soap and water were 0.03 times as likely to wear a mask (AOR 0.027, 95% CI 0.004-0.182). Despite the cost-effectiveness of frequent hand washing, the qualitative report included the following observation: “40% of students reported that lack of access to filled water containers at every corner regularly has been a challenge at the school.”

In addition, a 21-year-old male participant reported in an interview that “along with mask use, students used to protect themselves using other alternative mechanisms too.”

Students who had started to stay home were 0.168 times as likely to wear a mask (AOR 0.168, 95% CI 0.054-0.52) and those who agreed that COVID-19 is fatal were 0.236 times as likely (AOR 0.236, 95% CI 0.084-0.666). Students who agreed that the flu vaccine is sufficient for preventing COVID-19 were 3.9 times more likely to wear a mask than those who did not (AOR 3.874, 95% CI 1.540-9.749), and those who disinfected equipment and working areas in market areas at least once a day were 0.22 times as likely compared to those who did not (AOR 0.222, 95% CI 0.086-0.575; Table 4).

**Table 4:** Factors associated with mask use among students and associated factors at Gambella Teachers’ Education and Health Science College, Gambella Region, Southwest Ethiopia.

Variables and categories			COR^a^ (95% CI)		AOR^b^ (95% CI)		*P* value^c^
**Sex**							
	Male	1		1			
	Female	3.07 (1.52-6.23)^d^		3.31 (1.19-9.24)		.02	
**Age category (years)**							
	<20	2.01 (1.05-3.89)^d^		2.55 (1.15-5.62)		.009	
	20-25	0.41 (0.34-1.22)		0.27 (0.05-1.35)		.02	
	>25	1		1			
**Agreement that not all persons with COVID-19 develop severe disease**							
	Yes	2.67 (1.38-5.12)^d^		3.37 (1.35-8.40)		.009	
	No	1		1			
**Agreement that there is currently no effective cure**							
	Yes	3.42 (1.03-11.32)^d^		6.27 (1.35-28.98)		.02	
	No	1		1			
**Perform proper washing with soap and water**							
	Yes	0.25 (0.09-0.70)^d^		0.02 (.004-0.18)		<.001	
	No	1		1			
**Have started to stay home**							
	Yes	0.31 (0.14-0.69)^d^		0.16 (0.05-0.52)		.002	
	No	1		1			
**Agreement that COVID-19 is fatal**							
	Yes	0.24 (0.12-0.49)^d^		0.23 (0.08-0.66)		.006	
	No	1		1			
**Agreement that flu vaccine is sufficient for preventing COVID-19**							
	Yes	1		1			
	No	2.69 (1.45-4.99)^d^		3.87 (1.54-9.74)		.004	
**Disinfect equipment and working areas at least once a day**							
	Yes	0.57 (0.31-1.06)		0.22 (0.08-0.57)		.002	
	No	1		1			

^a^COR: crude odds ratio.

^b^AOR: adjusted odds ratio.

^c^*P*≤.05 was considered statistically significant.

^d^Candidates selected at *P*<.25.

## Discussion

### Principal Findings

This study investigated face mask use and associated factors among students at Gambella Teachers’ Education and Health Science College, Southwest Ethiopia, in 2021. The overall magnitude of face mask use was found to be 87% (330/379). Factors associated with mask use included being female and younger, agreeing that not all persons with COVID-19 develop severe disease, agreeing that there is currently no effective cure, performing proper washing with soap and water, having started to stay home, agreeing that COVID-19 is fatal, agreeing that flu vaccination is sufficient for preventing COVID-19, and disinfecting equipment and working areas at least once a day.

This study, conducted in Ethiopia, found that the magnitude of mask use among students was 87%, which is relatively lower than what was found by a study conducted in Japan; most respondents in that study showed a moderate or higher frequency of washing their hands or wearing a mask (both 96.4%) [17]; other studies found mask use magnitudes of 91% in Saudi Arabia [18], 95.2% in western Uganda [23], 90% in the United Arab Emirates [20], and 87.94% in China (among undergraduate students) [21]. However, our finding of an 87% use rate is higher than some other studies: 45% in Mizan Tepi, Ethiopia [19], 74.1% in the towns of Dessie and Kombolcha in Ethiopia [24], and 51.6% in Wuhan, China [22]. The differences in the magnitude of mask use might be due to differences in the level of awareness of respondents, strictness of adherence to recommendations and regulations, access to services, and differences in services.

The odds of mask use among female students were 3.3 times higher than among male students. This finding is consistent with studies conducted among Japanese university students and in reopened secondary schools in Gonder, Northern Ethiopia [17,19]. Similarly, a study conducted in China revealed that women showed significantly higher levels of positive attitudes than men; moreover, there was a positive correlation between attitude and practice [19,21,29].

This study found that younger students were 2.55 times more likely to use face masks than older students. A study from the Amhara region of Ethiopia found that younger age was associated with better knowledge of COVID-19 [30], which, in turn, could be justified as being the result of those with good knowledge of COVID-19 being more likely to practice prevention measures [19].

Students who understood that not all persons with COVID-19 develop severe disease were about 3.4 times more likely to use masks than those who did not understand this. This seems to contradict previous literature showing that people with comorbid illness or other chronic diseases, such as diabetes mellitus and hypertension, and who are older were more likely to develop severe disease [31,32]. While it is true that not all persons will reach a severe stage of the disease, it has continued to be recommended that the public use face masks in specific situations with a high risk of infection, such as crowded places, irrespective of the local epidemiological situation.

This study found that students who agreed that there is currently no effective cure for COVID-19 were 6.3 times more likely to use masks than those who said they believed that there is an effective cure. Despite tremendous efforts to improve vaccine availability, access, and quality to control COVID-19, the World Health Organization (WHO) continues to recommend the use of masks by the public in specific situations and updated this advice to recommend their use irrespective of the local epidemiological situation, given the current spread of COVID-19 globally. Masks also reduce disease severity at schools, where there is a great deal of social gathering and interaction [5,11].

The literature overwhelmingly recommends proper washing with soap and water along with other behavior to help prevent COVID-19 infection [22]. Despite this, our study found that respondents who washed frequently were 0.03 times as likely to use a mask. It could be the case that it is an optional behavior for students to wash frequently, whereas using face masks is not optional. Meanwhile, studies have reported that some students do not use masks due to financial and affordability issues across different settings [12].

Students who had started to stay home were 87% less likely to use face masks than who did not stay home. The reason for this could be that masks are used in places where people move and interact, which can expose them to infection. On the other hand, those who stay home usually practice recommended prevention methods other than mask use.

Students who agreed that COVID-19 is fatal were about 74% less likely to use masks than those who did not agree. A study from Debreberhan University found that students perceived low risks related to the COVID-19 pandemic at the time of school resumption [33]. Despite the low perception of the deadliness of COVID-19, the students might have been exposed to other preventive measures, like hand washing and social distancing [15]. Meanwhile, a Polish study among health care workers suffering from face mask–induced itchiness found that they were 31.6% less likely to comply with mask use [34], while another study found that most students supported the required use of masks in schools and indoor public spaces [35].

The odds of mask use among respondents who agreed that flu vaccination was sufficient for preventing COVID-19 were 3.9 times greater than among those who did not agree. This contrasts with a study from Saudi Arabia that found that 90.4% of students avoided contact with other people if they had flu-like symptoms [20]. On the other hand, about 50% of students rightly stated that antibiotics and flu vaccines are not effective against COVID-19 infection [36]. A recent study conducted by Kinyili et al [37] showed that an increased extent of mask-wearing among vaccinated individuals increased with an increasing level of vaccination and that regular face mask use resulted in a sharp decrease in COVID-19 infections. However, wearing face masks alone also resulted in a reduction in the peak number of infections with an increasing level of face mask efficacy, although there can be a delay as the infections are cleared; that is, treatment of COVID-19 does not always lead to immediate recovery, as mild to moderate symptoms persist [38].

The WHO recommends cleaning and disinfecting bathroom and toilet surfaces at least once daily using regular household soap or detergent, and then, after rinsing, applying regular household disinfectant [39]. This agrees with our finding that respondents who disinfected their equipment and working areas at least once a day were 78% less likely to use masks. Students who were effective in maintaining washing, disinfecting, and applying social distancing might not have used masks. Similarly, about 80% (16/20) of students reported that lack of access to masks and affordability of masks were restraints on their regular use after schools were reopened.

### Limitations

We made extensive efforts to reduce possible shortcomings of this study. Despite that, this study has certain limitations. First, the study was limited to students at a government college; therefore, there is a question of the representativeness of the findings to all students. In addition, the data presented in this study are self-reported and may thus be subject to recall bias. Furthermore, the study has a limited ability to determine cause-effect relationships because of its cross-sectional design.

### Conclusion

The overall magnitude of mask use of 87% (330/379) shows that mask use is relatively moderate in Ethiopia. Several factors, such as sex, age, having started to stay home, agreeing that not all persons with COVID-19 develop severe disease, agreeing that there is currently no effective cure, agreeing that proper washing with soap and water is effective, agreeing that COVID-19 is fatal, and agreeing that the flu vaccine is sufficient to prevent COVID-19 were independently associated with mask use among students. Therefore, the college should aggressively encourage students to wear masks, monitor the implementation of COVID-19 prevention regulations, and improve the accessibility of masks.

## Abbreviations

AOR: adjusted odds ratio
WHO: World Health Organization

## Data Availability

The data sets used and/or analyzed during the current study are all presented within the manuscript.
